# Microbial composition of egg component and its association with hatchability of laying hens

**DOI:** 10.3389/fmicb.2022.943097

**Published:** 2022-10-20

**Authors:** Jiaming Jin, Qianqian Zhou, Fangren Lan, Junying Li, Ning Yang, Congjiao Sun

**Affiliations:** Ministry of Agriculture and Rural Affairs, National Engineering Laboratory for Animal Breeding and Key Laboratory of Animal Genetics, Breeding, and Reproduction, China Agricultural University, Beijing, China

**Keywords:** microbial composition, egg yolk, egg white, embryonic development, microbiota, chicken eggs

## Abstract

The internal quality of eggs is critical for human consumption and embryonic development. However, microorganisms inside eggs have not been thoroughly investigated for their roles in determining the egg’s internal quality. Here, a total of 21 hens were selected from more than 1,000 chickens based on their hatching results and were divided into high- and low-hatchability groups. Then, we collected 72 eggs from these 21 hens to obtain egg whites and yolks, including 54 fresh eggs and 18 eggs after 12 days of incubation. We characterized the microbial composition of egg yolks and whites, the microbial change along incubation, and differences in microbial abundance between the high- and low-hatchability groups. The results indicated that egg whites are not sterile. Proteobacteria, Firmicutes, Actinobacteria, and Bacteroidetes were the dominant phyla in egg yolk and white. There was a large difference in the microbial composition between egg whites and yolks, and this difference increased after 12 days of incubation. Egg whites have lower microbial diversity than egg yolks owing to the presence of antibacterial substances such as lysozyme in the egg white. After a 12-day incubation, the microbial diversity decreased in egg whites but increased slightly in egg yolks. Meanwhile, the microbes in egg white can migrate to egg yolk during incubation. Additionally, Genus *Muribaculaceae* was identified as a biomarker in egg yolks incubated for 12 days and was more often detected in healthy groups. On the contrary, more genus *Rothia* were found in the fresh egg yolk of the low hatchability groups and was considered to have low virulence. These findings shed light on the composition and differences in microbiota between egg yolks and whites and may open new avenues for studying embryonic development in chickens.

## Introduction

An egg is a biological system intended to ensure the health of the embryo and allow it to successfully hatch into a chicken (Wilson, 1997; Moran, 2007; Liu et al., 2018). During incubation, eggs provide nutrients and other necessities for the growth and development of the embryo. However, not every embryo will successfully develop into a chick. Factors affecting embryonic growth include maternal effects (breed, age, and maternal nutrition status) (King’Ori, 2011), rooster semen quality, incubation conditions (such as temperature, humidity, light, and ventilation), and egg quality (including egg weight, eggshell thickness, porosity, and shape index) (Heier and Jarp, 2001). In production, large variances in hatchability among chickens are frequently observed, even if the hens were from the same breed, of the same age, raised in the same environment, and if their fertile eggs have a similar quality (Wang et al., 2019). Therefore, the influence of egg internal constituents on hatchability must be considered.

The growth and development of chicken embryos rely on the essential amino acids, lipids, carbohydrates, and minerals stored in eggs (Narushin and Romanov, 2002; Vieira, 2007; Ho et al., 2011; Yadgary and Uni, 2012; van der Wagt et al., 2020). The egg yolk is the main source of nutrients for embryo growth and it influences embryo viability (Peebles et al., 2000). The egg white mainly plays a role in resisting bacterial invasion and provides nutrients to the embryo, which has been reported as necessary for the start of embryo development (Willems et al., 2014). Several studies have confirmed that certain functional proteins in eggs can influence hatchability (Muramatsu et al., 1990; Rehault-Godbert et al., 2014; Cheng et al., 2021). Other studies in mammals have demonstrated that microbiota from various maternal sites during pregnancy may potentially influence the health and passive immunity of offspring. The placental microbiota affects pregnancy outcomes (Pelzer et al., 2017; Nyangahu and Jaspan, 2019), and plays a yet unknown role in early embryonic development (Koren et al., 2012; Wen et al., 2021).

Initially, the egg white was considered sterile. However, a recent study discovered microorganisms inside the egg white (Lee et al., 2019). The presence of microorganisms has also been confirmed in egg yolks (Ding et al., 2022). Eggs are formed in the maternal reproductive tract, and the embryo in the egg grows and develops into a chick after 21 days of incubation. Previous research confirmed the presence of microorganisms in the maternal reproductive tract and digestive tracts of 1-day-old chicks (Pajurek et al., 2019; Zhou et al., 2021). Several studies have shown that hen gut and fecal microbes are associated with both egg formation and fertility (Elokil et al., 2020a,b). This suggests that microbes may have an effect on the reproductive traits or eggs of the hens. Although it has become clear that the egg yolk and white are not sterile, the microbial compositions of egg yolks and whites in fresh and hatching eggs, and their influence on hatchability, remain unknown.

Hence, in the current study, we performed 16S rRNA gene sequencing on 144 egg white and yolk samples to characterize the microbial composition of fresh and incubated eggs. Additionally, we compared the differences between egg whites and yolks and analyzed the changes in microbial composition along with egg incubation. We further identified embryo growth-related microorganisms that were differentially represented between the high and low hatchability groups. The findings of this study will expand our understanding of the microbial composition of eggs and provide additional insights into embryo development.

## Methods and materials

### Ethics statement

The protocol was approved by the Animal Care and Use Committee of China Agricultural University (Permit Number: AW08059102–1).

### Animal selection and sample collection

**FIGURE 1:**
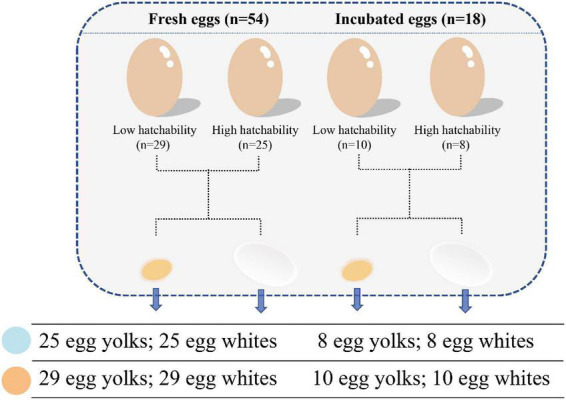
Scheme of sample collection including egg compartment, incubation, and hatchability.

A pure line derived from Rhode Island Red chickens from Beijing Huadu Yukou Poultry Industry Co., Ltd., was used in this study. This population consisted of 90 males and 1,011 females. All 1,011 hens were artificially inseminated (male/female ratio = 1:11–12) to form 90 rooster families. Each hen produced an average of 17 fertilized eggs, which were incubated under the same conditions to record the hatchability data. Based on the hatchability data, 11 hens with hatchability of less than 60% from 11 different rooster families were selected to form the low hatchability group (LH, <60%). To eliminate the effect of sperm quality, 11 hens with a hatching rate close to 100% (HH, ∼100%) were selected from the same families as the individuals in the LH group and with egg quality comparable to that of the LH group. All roosters and hens selected for the trial were in good health. Fresh unfertilized eggs were then collected for three continuous days from these two groups at 42 weeks of age, and one hen was removed from the LH group after 3 days of no egg collection. During sampling, we ensured that the environment was strictly sterile. Fresh egg yolks and mixed egg whites were collected and stored in 2 ml cryotubes (Axygen) and placed in liquid nitrogen at once and then stored at −80°C until usage. Thereafter, each hen was inseminated with the mixed sperm of five roosters to collect fertilized eggs for another 3 days. Before incubation, the surfaces of the fertilized eggs were disinfected. The incubator (EIFDMS-16800) and its corresponding room had been sanitized by fumigation (40% formaldehyde solution) to ensure a sterile environment. After the eggs are put into the incubator, 14 g of potassium permanganate and 28 ml of formaldehyde are used for fumigation per cubic meter for 20 min. The egg white and yolk samples were collected after 12 days of incubation. In total, we obtained 144 egg white and yolk samples from 54 fresh and 18 incubated eggs that could be assigned to high and low hatchability groups (Figure 1).

### DNA extraction and 16S rRNA genes sequencing

Genomic DNA was extracted using an OMEGA Soil DNA Kit (M5635-02) (Omega Bio-Tek, Norcross, GA, USA) according to the manufacturer’s instructions. DNA quality was assessed using 1.2% agarose gel electrophoresis (Invitrogen), and a NanoDrop NC2000 spectrophotometer (Thermo Fisher Scientific, Waltham, MA, USA) was used to measure the concentration and purity of extracted DNAs.

Polymerase chain reaction (PCR) amplification of the V4 hypervariable region of the bacterial 16S rRNA genes was performed using the primer pair 515F (5’-GTGCCAGCMGCCGCGGTAA-3’) and 806R (5’-GACTACHVGGGTWTCTAAT-3’) (Shterzer et al., 2020) on an Applied Biosystems 2720 Thermal Cycler (ABI). The PCR reaction volume contained 5 μl of buffer (5×), 5 μl of GC buffer (5), 0.25 μl of Q5^®^ High-Fidelity DNA Polymerase (NEB, 5 U/μl), 5 μl of dNTPs (2.5 mM), 1 μl each of forward and reverse primer (10 μM), 2 μl of DNA template, and 8.75 μl of ddH_2_O. Thermal cycling was performed as follows: 98°C for 2 min, 98°C for 15 s, 55°C for 30 s, 72°C for 30 s, 72°C for 5 min, and held at 10°C for a total of 25–30 cycles. PCR products were purified using Vazyme VAHTSTM DNA Clean Beads (Vazyme, Nanjing, China) and quantified using the Quant-iT PicoGreen dsDNA Assay Kit (Invitrogen, Carlsbad, CA, USA). After individual quantification, the barcoded V4 amplicons were pooled and analyzed on an Illumina NovaSeq platform with NovaSeq 6000 SP Reagent Kit (500 cycles) (Illumina) and sequenced as paired-end 250 base pair (bp) read lengths at Shanghai Personal Biotechnology Co., Ltd (Shanghai, China). Some of the data were analyzed using the Personal GenesCloud online platform.^1^

### Bioinformatics assessment

QIIME2 2019.4 (Bokulich et al., 2013) was used for demultiplexing, filtering, denoising, merging, and removing chimeras of the raw sequence data. High-quality sequences were aligned and clustered to generate a count table of amplicon sequence variants (ASVs) with 100% identity (Callahan et al., 2017). The taxonomy was then assigned against the Silva database (Release132) using a pre-trained naive Bayes classifier and q2 feature-classifier plugin (Zaura et al., 2009). ASV filtration can improve diversity estimates in all taxa. ASVs with a relative abundance >10^–6^ were selected for retention (Bokulich et al., 2013; Benjamino et al., 2018). Finally, 7,924 ASVs remained and were classified at the domain, phylum, class, order, family, genus, and species levels.

Subsequent analyses were based on Qiime2 (Bolyen et al., 2019). A rarefaction curve was generated to investigate the depth of sequencing, using a maximum rarefaction depth of 10,000 sequences and the observed ASV index (Supplementary Figure 1). Rarefied ASV data were used to calculate the α-diversity metrics of the Shannon and Simpson indices using the vegan package (Oksanen et al., 2013) in R (v4.0.2).

### Statistical analysis

Differences in hatchability and egg quality traits were examined using *t*-tests in R (v4.0.2) (R Core Team, 2013). The different groups were statistically compared through the analysis of similarity (ANOSIM) with 999 permutations (Clarke, 1993). Venn diagrams were constructed using the *R* (v4.0.2) package “VennDiagram” at the ASV level (Chen and Boutros, 2011). Random forest was used to identify the microbes enriched in different groups and to order the microbes according to their importance (Breiman, 2001). For the verification of the accuracy of the prediction classification model of random forest, we performed the receiver operating characteristic (ROC) curve to test the sensitivity and accuracy of the model. Linear discriminant analysis effect size (LEfSe) was used to compare significant differential bacteria across groups (Segata et al., 2011). Relative abundance was converted to percentages for this analysis. The threshold on the current linear discriminant analysis (LDA) score for discriminative features was set at 4.0 and 2.0 in the comparisons of different groups. Wilcox rank-sum test was used to analyze the differences between groups using the stats package (R Core Team, 2013) in R (v4.0.2). The Duncan test was used for the multiple test method. The *Post hoc* test slasher significant level value is set to 0.95 and adjusted with the BH method (Signorell et al., 2019). Principal coordinate analysis (PCoA) based on beta diversity across samples was performed using the Bray-Curtis distance metrics in Qiime2 (Bolyen et al., 2019).

## Results

### Classification and composition of the microbiota in egg white and yolk

**FIGURE 2:**
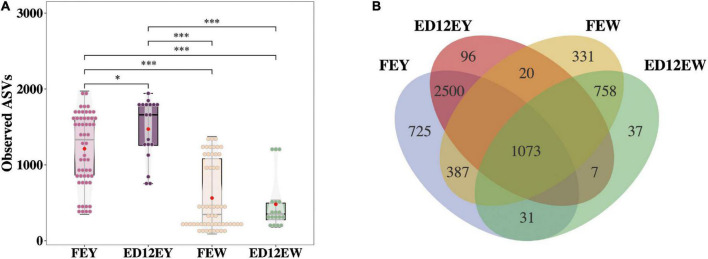
The number of amplicon sequence variants (ASVs) and the shared ASVs in different groups. **(A)** The boxplot depicts the total number of ASVs detected in each group. **(B)** A Venn diagram demonstrates the core ASVs among four groups. *P < 0.05, ***P < 0.001, and ns means no significance.

Hens in the high- and low- hatchability groups had significant differences in hatchability (*p* < 0.01, T- statistic), but no significant difference was observed for egg quality traits which could influence hatchability to some extent (*p* > 0.05), and this made the subsequent analyses more meaningful (Table 1). To characterize the microbial composition of the eggs, egg white and yolk samples from 54 fresh eggs (FE) and 18 fertilized eggs incubated for 12 d (ED12) were collected. The microbiota was detected in all samples. A total of 13,961,188 quality-filtered sequences were obtained from 144 samples, with an average of 96,953 reads (Supplementary Table 1). These sequences finally clustered into 7,924 ASVs. The number of ASVs detected in the yolks of fresh and incubated eggs was greater than that in the egg whites (*p* < 0.001, W-statistic) (Figure 2A). The ASVs detected in the yolks of fresh and incubated eggs differed significantly (*p*_adj_ < 0.05, W-statistic, *Post hoc* test), and this difference was greater than that in the egg whites (*p*_adj_ > 0.05, W-statistic, *Post hoc* test). The Venn diagram shows the number of shared ASVs detected in each group, and there was a 20.06% (2,500) and 40.88% (758) overlap between egg yolks and whites of FE and ED12, respectively (Figure 2B). Subsequently, the microbiota presented in egg whites was classified into 49 phyla, 522 families, 1,226 genera, and 2,233 species, whereas egg yolk microbiota was classified into 51 phyla, 533 families, 1,311 genera, and 2,558 species.

**TABLE 1:** Hatchability, fertility, eggshell strength, and egg weight between high and low hatchability groups.

Group	Hatchability (%)	Fertility (%)	ESS (kg/cm^2^)	EW (g)
High hatchability	99.5 ± 1.44^a^	91.92 ± 7.31	3.79 ± 0.45	57.29 ± 3.71
Low hatchability	45.54 ± 11.31^b^	91.5 ± 7.2	3.91 ± 0.47	58.65 ± 4.1

^a,b^Means values with no common superscripts within each row differ significantly (*P* < 0.05) when tested with *T*-tests. All values are shown as mean ± SD.

### Microbial differences between egg yolk and egg white

**FIGURE 3:**
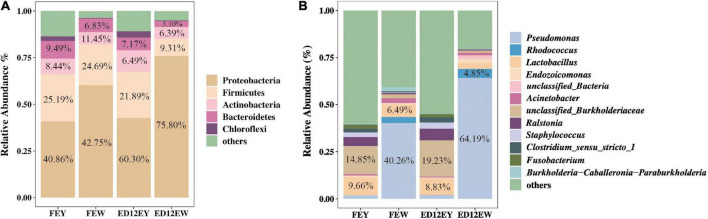
Microbiota composition in egg yolk and egg white for fresh and incubated eggs. **(A)** The relative abundance of dominant microbial phyla in the egg yolks and whites at different periods. **(B)** The relative abundance of 12 genera in the egg yolks and whites at different periods. 12 genera were comprised of the top five genera with the most relative abundance without overlap in each group.

At the phylum level, egg yolks and whites had similar dominant microbial communities, including Proteobacteria (∼54.93%), Firmicutes (∼20.27%), Bacteroidetes (∼6.65%), and Actinobacteria (∼8.19%). Proteobacteria and Firmicutes were the two most abundant phyla, with a combined relative abundance of > 65% in the four groups (Figure 3A and Table 2). Proteobacteria were more abundant in egg whites (FEW 60.30%, ED12EW 75.8%) than in yolks (FEY 40.86%, ED12EW 42.75%), regardless of whether the eggs were fresh or incubated. The remaining three dominant phyla had lower relative abundances in egg whites than in egg yolks in fresh or incubated eggs (Supplementary Figure 2). We also observed a considerable increase in the proportion of Proteobacteria with incubation, as well as a decrease in the abundance of Firmicutes (FEW 21.89%, ED12EW 9.31%) and Bacteroidetes (FEW 7.17%%, ED12EW 3.10%) at the phylum level between FEW and ED12EW, whereas these phyla between FEY and ED12EY remained largely stable.

**TABLE 2:** Relative abundance of microbial phyla in egg yolks and whites at different periods.

Phylum	FEY	ED12EY	FEW	ED12EW
Proteobacteria	40.86%	42.75%	60.30%	75.80%
Firmicutes	25.19%	24.69%	21.89%	9.31%
Actinobacteria	8.44%	11.45%	6.49%	6.39%
Bacteroidetes	9.49%	6.83%	7.17%	3.10%
Chloroflexi	2.42%	3.42%	0.47%	0.33%
Others	13.60%	10.87%	3.67%	5.06%

At the genus level, most of the microbiota had a low relative abundance, with an average relative abundance of 2%. The microbial composition of egg whites and egg yolks differed greatly. An unclassified genus in the Burkholderiaceae family was predominant, with a relative abundance of 14.85%, followed by *Lactobacillus* (9.66%) in the egg yolks of the two groups. *Pseudomonas* with a relative abundance greater than 40%, was the most dominant genus in the egg whites (Figure 3B and Supplementary Table 2). After incubation, the egg yolk microbiota remained stable; however, the egg white microbiota gradually shifted from a predominance of *Pseudomonas* (FEY 40.26%) and *Lactobacillus* (FEY 6.49%) to *Pseudomonas* (ED12EY 64.19%) and *Rhodococcus* (ED12EY 4.85%). The remaining dominant genera all experienced a decrease in relative abundance following incubation (Supplementary Figure 3). Additionally, 29.6% of all genera were found to decrease in relative abundance with incubation in egg whites, while the abundance of these genera increased in egg yolks with incubation (Supplementary Tables 3, 4).

### Microbiota diversity of egg white reduced after incubation

**FIGURE 4:**
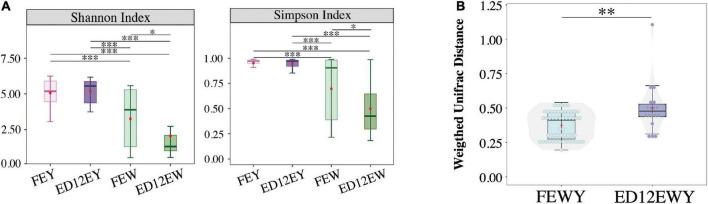
Microbial diversity of each group. **(A)** Alpha-diversity (Shannon and Simpson indexes) of microbiota in four groups exhibiting community diversity. **(B)** The changes between egg yolks and whites on Microbial weighted UniFrac distance. FEWY represents fresh egg yolks and whites and ED12EWY represents egg yolks and whites at 12 embryonic ages. *P < 0.05, **P < 0.01, ***P < 0.001.

The changes of Shannon and Simpson Indices both revealed that the microbiota diversity in egg white significantly decreased (*p*_adj_ < 0.05, W-statistic, *Post hoc* test) after incubation (Figure 4A), whereas the diversity in egg yolk had a trend of increasing (*p*_adj_ > 0.05, W-statistic, *Post hoc* test). Meanwhile, microbes in egg white maybe unstable. ANOSIM showed that FEW had a closer Bray-Curtis distance with ED12EY than with FEY (FEW-FEY, R = 0.543, R statistic; FEW-ED12EY, R = 0.418, R statistic) (Supplementary Figure 4 and Supplementary Table 5).

Weighted UniFrac distance was performed to demonstrate whether there was variation in the microbial community of the egg yolk and white during incubation. The boxplot shows that egg yolk and egg white of ED12 eggs (ED12YW) had a greater weighted UniFrac distance (*p* < 0.05, W-statistic) than fresh egg yolk and white (FEYW) (Figure 4B). As a result, fresh egg yolk and white had a higher microbial similarity, which decreased with incubation.

### Identification of differential microorganisms in egg white and yolk before and after incubation

**FIGURE 5:**
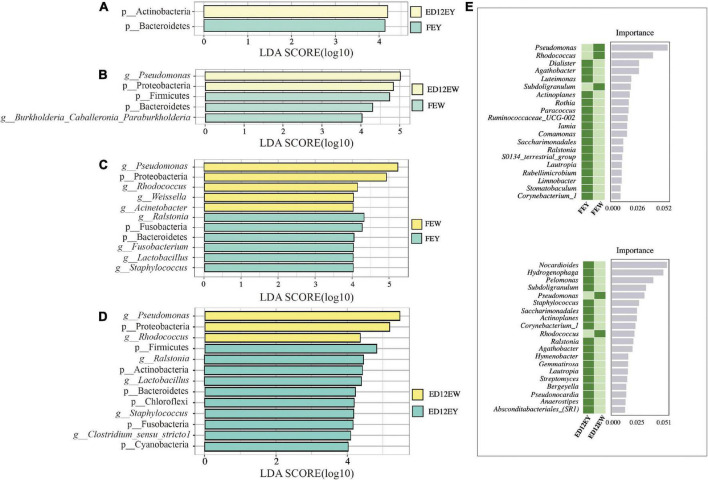
LEfSe results for the egg white and yolk. LEfSe analysis was used with an LDA score > 4.0 to show differential enrichment of the bacterial features as biomarkers between egg yolks and whites in different groups. **(A,B)** LEfSe shows the differential microbiota between fresh egg yolks and whites. **(C,D)** LEfSe shows the differential microbiota between egg yolks and whites at 12 embryonic ages. **(E)** Random forest analysis identifies the key differential genera between egg yolks and whites at different periods.

To examine the significantly different microbes between egg yolks and egg whites, we used LEfSe analysis and set an LDA score of 4.0 as the cutoff. The result of LEfSe showed that egg yolk and white microbiota altered with incubation. The phyla Bacteroidetes and Actinobacteria were identified as significantly representative taxa in FEY and ED12EY, respectively (Figure 5A and Supplementary Table 6). For FEW, the genus *Burkholderia-Caballeronia-Paraburkholderia* and phylum Firmicutes were the most significant differential taxa (Figure 5B and Supplementary Table 7). Interestingly, *Bacteroidetes* was also a significant differential genus in FEW. Meanwhile, the phylum Proteobacteria and the genus *Pseudomonas* were biomarkers of ED12EW. In the comparison of egg white and yolk microbes before and after incubation, the phylum Proteobacteria and the genus *Psedomonas* and *Rhodococcus* could be seen as significantly representative microorganisms both in fresh and incubated egg white (Figures 5C,D). The significantly representative microorganisms in egg yolk were the phylum Fusobacteria and Bacteroidetes and the genus *Ralstonia*, *Lactobacillus*, and *Staphylococcus*. Besides these overlapping microbes in egg yolk or white, each group also had its own enriched microorganisms at the phylum and genus levels. The genus *Weissella* and *Acinetobacter* were two genera only enriched in fresh egg whites. For ED12EY, the phyla Firmicutes, Actinobacteria, Chloroflexi, and Cyanobacteria, and the genus *Clostridium sensu stricto 1* were identified to be representative microbiota. The genus *Fusobacterium* was the only one taxa that significantly enriched in FEY.

To further define the distinctions between egg yolks and whites, a random forest analysis was used to identify the key differential genera. Between FEY and FEW, *Pseudomonas* was the most important representative genus with an average relative abundance of 40.26% in FEW and just 1.84% in FEY (Figure 5E and Supplementary Figure 5). *Nocardioides* was the most significant genus in ED12EY with relative abundances of 0.32%, and the abundance in ED12EW was 0.019% (Supplementary Table 8). The above two key genera were also detected by LEfSe analysis as differential microbes. We also performed a random forest analysis between FEW and ED12EW, and the abundance of key differential microbiota was very low, as well as the two egg yolk groups (Supplementary Figure 6).

### Screening of key hatchability-associated microorganisms

Based on hatchability, the samples were divided into four groups: egg yolks of low and high hatchability groups (LHEY/HHEY) and egg whites of low and high hatchability groups (LHEW/HHEW). The results suggested that the incubation influenced the microbial composition of the egg yolk and white. Therefore, we increased the original four groups to eight groups, i.e., fresh egg yolks of low and high hatchability groups (F-LHEY/F-HHEY), fresh egg whites of low and high hatchability groups (F-LHEW/F-HHEW), egg yolks incubated for 12 days in the low and high hatchability groups (ED12-LHEY/ED12-HHEY), and egg whites incubated for 12 days in the low and high hatchability groups (ED12-LHEW/ED12-HHEW).

**FIGURE 6:**
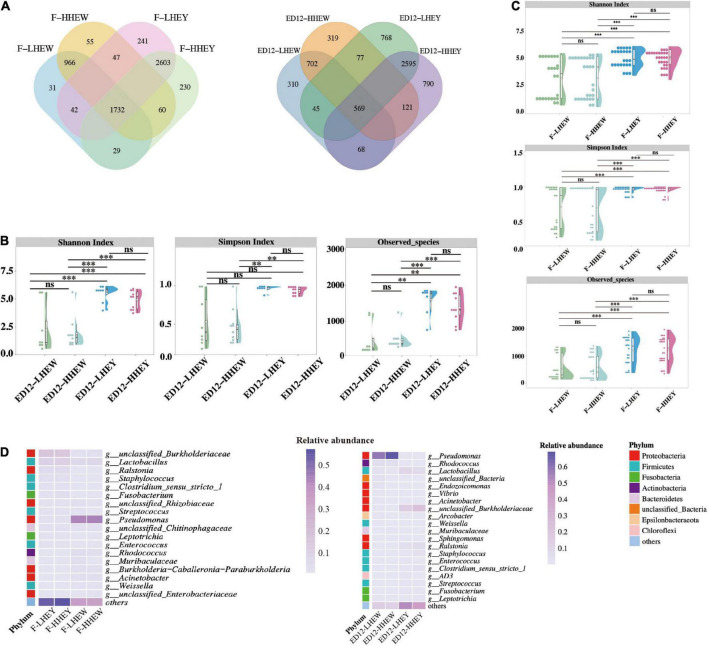
Microbial composition and diversity in egg white and yolk of low and high hatchability groups. **(A)** Overlap analysis of the shared amplicon sequence variants (ASVs) in high and low hatchability groups of egg yolk and white in fresh eggs and eggs for 12 days of incubation. **(B)** Alpha diversity (Shannon, Simpson, and Observed species) of F-LHEY, F-HHEY, F-LHEW, and F-HHEW. **(C)** Alpha diversity (Shannon, Simpson, and Observed species) of ED12-LHEY, ED12-HHEY, ED12-LHEW, and ED12-HHEW. **(D)** The heat map shows the results of the top 19 relative abundance of genera and 21 relative abundance of genera in four groups of fresh eggs and eggs at 12 embryonic ages. The 19 and 21 genera were obtained by collecting the top 10 relatively abundant genera that did not overlap in four groups at the same period. *P < 0.05, ***P < 0.001, and ns means no significance.

We explored the distribution of the ASV amounts in the high and low hatchability groups using Venn and violin diagrams (Figure 6A and Supplementary Figure 7). The two groups of LHEY (F-LHEY 5638, ED12-LHEY 3976) had obviously more ASVs than LHEW (F-LHEW 3381, ED12-LHEW 1774). The α-diversity of the microbiota was calculated in the eight groups to indicate the overall evenness and richness, using the Shannon and Simpson indices, and the observed species, respectively (Figures 6B,C). No significant difference was observed in egg yolks and whites between the high and low hatchability groups. In addition, there were no discernible differences in genera and phyla abundances between the high and low hatchability groups (Figure 6D and Supplementary Tables 9, 10). The results of PCoA and ANOSIM based on Bray-Curtis dissimilarity were similar (Supplementary Figure 8).

To further reveal the key microbes, the Wilcoxon rank-sum test and LEfSe were both applied to identify the differential enrichment of microorganisms at the genus level between high and low hatchability groups (Supplementary Tables 11, 12). In this way, we obtained several microbes with a high call rate and relative abundance as the main markers. However, some of these microbes have a relative abundance lower than 0.1% and were not considered crucial ones. Finally, the genus *Rothia* had greater abundance in F-LHEY than in F-HHEY, and the genus *Sphingomonas* was significantly higher presented in ED12-HHEW than ED12-LHEW. *Muribaculaceae* was the only key genus with higher abundance in the high-hatchability group (ED12-HHEY). Four differential genera with greater abundance in ED12-LHEY were the Subgroup_2 genus of the class Acidobacteriia, *JG30-KF-CM45*, *Rubrobacter*, and *Solirubrobacter*.

## Discussion

Poultry eggs as a key part of the human diet are consumed worldwide (Willems et al., 2014; Eddin et al., 2019). Consumers are becoming more concerned about egg quality, which is linked to food safety (Hisasaga et al., 2020). On the one hand, eggs can be a vehicle for the transmission of pathogens causing foodborne illnesses, such as *Salmonella* and *Escherichia coli* (Yin et al., 2013; Ehuwa et al., 2021; Mueller and Tainter, 2022). On the other hand, eggs can also act as a nexus to spread microbiota from the hen to the chick (Zhang et al., 2017; Dai et al., 2022). Previous studies have found that most albumen migration starts around ED 13 (Moran, 2007). The remaining albumen is mixed with amniotic fluid and swallowed by chicken embryos after ED 13 (Beulens et al., 2005). Hence, we selected eggs in two states for our study: fresh eggs and eggs that had been incubated for 12 days. Our study demonstrated that egg yolk and white are not free from microorganisms, in agreement with our earlier claim that eggs are not formed in a sterile environment (Wen et al., 2021). Similarly, we previously characterized the gut microbiota of 1-day-old chicks (Zhou et al., 2021). Therefore, one of the aims of this study was to fill the gap between studies on the microbiota in hens and chicks. To reduce the affect of environmental contaminants, setting a negative control, of course, is the best strategy for contaminants removal. The current study lacked negative control, but carried out many other measures. The surface of the eggs was sterilized. The incubator and its corresponding room were sanitized in advance to ensure the sterile environment, and the experimental materials including tubes and micro pipette tips were also disinfected. In addition, all operations such as egg white collection and DNA extraction were performed in the sterile fume hood. Furthermore, according to our results, we mainly focused on the microbes with relatively high abundance, trace of environmental microorganisms was hard to affect the results.

We characterized the microbiota of egg yolks and whites of chickens. As expected, the number of ASVs in egg yolks was greater than that in egg whites, supporting the conclusion that egg whites have antimicrobial properties, including the obstructive effect of ovomucin on microbial movement and the inhibition of microorganisms by functional proteins and alkaline environments in egg whites (Gantois et al., 2009; Paulson et al., 2013; Legros et al., 2021). Compared with egg yolk, egg white contains a less diverse microbial community. Similarly, the magnum, the site for egg white formation, also has very low microbial diversity due to the secretion of lysozyme and other antimicrobial proteins (Romanoff and Romanoff, 1967; Sah and Mishra, 2018). It can be speculated that during the formation of egg whites, a portion of the hen’s microbiota and antimicrobial substances are transferred to egg whites. Some studies have shown that microbial diversity may change at different sites in the oviduct (Edwards et al., 1976; Lee et al., 2019). Studies have shown that as the chicken embryo develops from ED 3 to ED 12, the number of gut microbes increases (Akinyemi et al., 2020). This is consistent with changes in egg yolk microbes in our study, however, it is unclear how the two are related. In terms of microbial composition, our analyses revealed that more than 80% of the microbiota was composed of the phyla Proteobacteria, Firmicutes, Actinobacteria, and Bacteroidetes, with Proteobacteria being the most abundant in each group, which echoes previous studies of the embryonic intestine (Ding et al., 2017). Microorganisms of the phylum Proteobacteria are generally regarded as pathogenic to humans (Chow and Lee, 2006; Rizzatti et al., 2017). Nevertheless, some hosts of non-disease states can also have a high ratio of Proteobacteria (Guaraldi and Salvatori, 2012), suggesting that some Proteobacteria bacteria may have additional functions in egg whites. The majority of microorganisms are facultative anaerobes owing to the presence of oxygen in the eggs. By absorbing oxygen, modifying the pH, and lowering the redox potential, facultative bacteria such as some genera of Proteobacteria provide favorable conditions for anaerobic bacterial colonization (Chow and Lee, 2006). The oxygen consumption by aerobic bacteria creates an anaerobic environment that promotes the growth and colonization of anaerobic bacteria in the intestinal tract of chicks (Wall et al., 2009; Jha et al., 2019). Evidence suggests that the chicken gut is first colonized by facultative anaerobic bacteria and subsequently replaced by anaerobic bacteria (Awad et al., 2016).

The alpha diversity of egg whites decreased throughout incubation, resulting in a more monolithic microbial composition. Egg white contains a variety of antimicrobial compounds, and the outer and inner eggshell membranes contain traces of antibacterial molecules that inhibit bacterial growth and migration (Gast and Holt, 2000; Hincke et al., 2000). Conversely, a slight increase in microbial richness was observed in the egg yolks from FE to ED12. The result of ANOSIM showed the composition of FEW shared a higher similarity with ED12EY than with FEY. A total of 423 genera were also found in egg white that the relative abundance decreased with incubation, at the same time, the relative abundance of these in the egg yolk increased from FEY to ED12EY. It can be deduced that the migration of the microbiota from the egg white to the egg yolk provides bacteria with access to a pool of nutrients. Consequently, there was rapid growth at an appropriate temperature during incubation. This may lead to an increase in the microbial diversity of the egg yolk after 12 days of incubation.

To ascertain the difference in microbial composition between egg yolks and whites, we conducted a simultaneous differential analysis of microorganisms at all taxonomic levels. As shown by LEfSe, ED12EY contained a higher abundance of the phylum Actinobacteria, with a detection rate of 100%, than that of FEY. In the phylum Actinobacteria, *Rhodococcus* was the most prevalent genus. *Rhodococcus* is not only a source of useful enzymes, but also possesses a diverse range of metabolic capabilities, including the breakdown of toxic chemicals, absorption, and production of applicable compounds (Martinkova et al., 2009; Thomas et al., 2011). Bacteroidetes were more abundant in the FEY and FEW groups, and more abundant in egg yolk than in egg white. Bacteroidetes are well-known for their role in the breakdown of biopolymers and carbohydrates, particularly polysaccharides, in mammals (Chevalier et al., 2017; Ding et al., 2017). Microbiota requires nutrients from the egg yolk to survive, but the remaining nutrients are depleted with incubation, resulting in a decrease in microbe abundance, such as *Bacteroidetes*. In ED12EW, the *Pseudomonas* genus and its corresponding phylum Proteobacteria had a higher relative abundance. The *Pseudomonas* genus is well-known for its metabolic versatility (Tsujii et al., 2007), and ED12EY exhibits this feature to a greater extent.

*Rothia* was identified as a significantly different microbe (*p* < 0.05) between F-LHEY and F-HHEY. The species of *Rothia* present in human hosts are thought to have low virulence and are increasingly recognized as opportunistic human pathogens, mainly causing a range of serious infections in immunocompromised hosts (Maraki and Papadakis, 2015; Fatahi-Bafghi, 2021). In this study, the genus *Rothia* was upregulated in F-LHEY, and we propose that its virulence would play a role when the host decreases in immunity and could cause the embryo to die during incubation. Notably, *Muribaculaceae* was the only key microbe that was more prevalent in the high hatchability groups of ED12-LHEY and ED12-HHEY. In the Wilcoxon test, the key microbe between ED12-LHEW and ED12-HHEW was more prevalent in ED12-HHEW. Early microbial research in animal guts and feces reported that the phylum or family of *Muribaculaceae* mainly has positive functions in the hosts. Genes involved in carbohydrate metabolism were upregulated in the Muribaculaceae family in the mouse gut (Chung et al., 2020). In many studies, the abundance of *Muribaculaceae* increased in healthy control groups and decreased in disease experimental groups (Evans et al., 2014; Bäuerl et al., 2018). Thus, *Muribaculaceae* may play a beneficial role in host health. Other studies have proved this conjecture to some extent. An earlier study showed that the relative abundance of the *Muribaculaceae* family was lower in elderly mice than in young mice (Shenghua et al., 2020). Moreover, a higher relative abundance of the *Muribaculaceae* family was correlated with an extended lifespan (Smith et al., 2019) and *Muribaculaceae* was also found to have a high relative abundance in the gut of long-living rodent *Spalax leucodon* (Sibai et al., 2020). Overall, *Muribaculaceae* were considered to play a positive role in the high-hatchability groups.

## Conclusion

The current study performed the 16S rRNA gene sequencing of the microbiota in egg whites and yolks and demonstrated the composition and differences in microbial abundance in egg yolks and whites, as well as the effect of incubation on the microbial community. The microbial diversity in egg white was lower than that in egg yolk, and the microbial composition of the egg white was more single after 12 days of incubation. *Rothia* and *Muribaculaceae* were the genera that played an important role in the hatching rate. These observations may shed new light on the microbial composition of eggs and search for hatching-related factors. It also serves as a link between maternal, embryonic, and chick microbial studies.

## Data availability statement

The datasets presented in this study can be found in online repositories. The name of the repository and accession number can be found below: NCBI Sequence Read Archive under BioProject ID PRJNA837337.

## Author contributions

CS and NY designed the study. JJ, FL, and QZ collected the samples. JJ performed the analysis and wrote the manuscript. FL and QZ assisted in data analyzing. JL, NY, and CS contributed to the revisions. All authors read and approved the final manuscript.

## Footnotes

1
https://www.genescloud.cn

## Supplementary material

Supplementary Figure 1 Rarefaction curves of FEY, FEW, ED12EY, and ED12EW.

Supplementary Figure 2 **(A)** The alluvial diagram shows the results of the top 5 relative abundance of phyla in FEY and ED12EY. **(B)** The alluvial diagram shows the results of the top 5 relative abundance of phyla in FEW and ED12EW.

Supplementary Figure 3 **(A)** The alluvial diagram shows the results of the top 11 relative abundance of genera in four groups. The 11 genera were obtained by collecting the top 10 relatively abundance genera that did not overlap in FEY and ED12EY. **(B)** The alluvial diagram shows the results of the top 14 relative abundance of genera in four groups. The 14 genera were obtained by collecting the top 10 relatively abundance genera that did not overlap in FEW and ED12EW.

Supplementary Figure 4 **(A)** ANOSIM analysis between FEW and FEY. **(B)** ANOSIM analysis between FEW and ED12EY.

Supplementary Figure 5 Receiver operating characteristic (ROC) curve of Random Forest.

Supplementary Figure 6 **(A)** Random forest analysis identifies the key differential genera between FEY and ED12EY. **(B)** Random forest analysis identifies the key differential genera between FEW and ED12EW.

Supplementary Figure 7 Differences in the number of ASVs among egg yolks and eggs in high and low hatchability groups in fresh eggs and eggs for 12 days of incubation.

Supplementary Figure 8 **(A)** Principal coordinate analysis (PCoA) plot with ANOSIM table showed the similarity in the microbial composition of four groups of fresh eggs. **(B)** Principal coordinate analysis (PCoA) plot with ANOSIM table showed the similarity in the microbial composition of four groups of eggs after 12 days of incubation.
