# Supplementary figures and images for: Microbial composition of egg component and its association with hatchability of laying hens
Source: Front Microbiol. 2022 Oct 20;13:943097. doi: 10.3389/fmicb.2022.943097 (PMC9632351; doi:10.3389/fmicb.2022.943097)

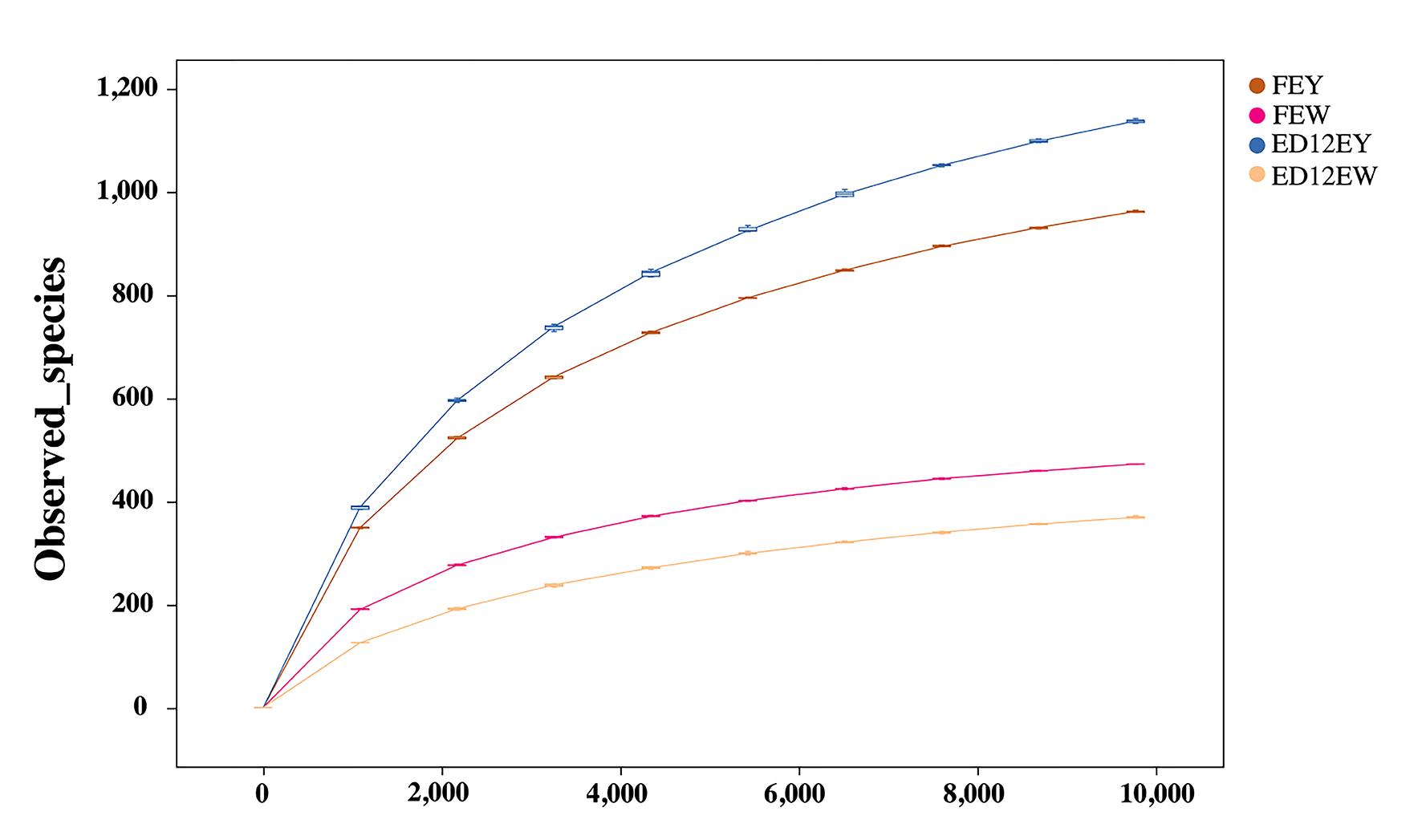

Supplement: Supplementary Figure 1 — Rarefaction curves of FEY, FEW, ED12EY, and ED12EW. [file Image_1.TIF]

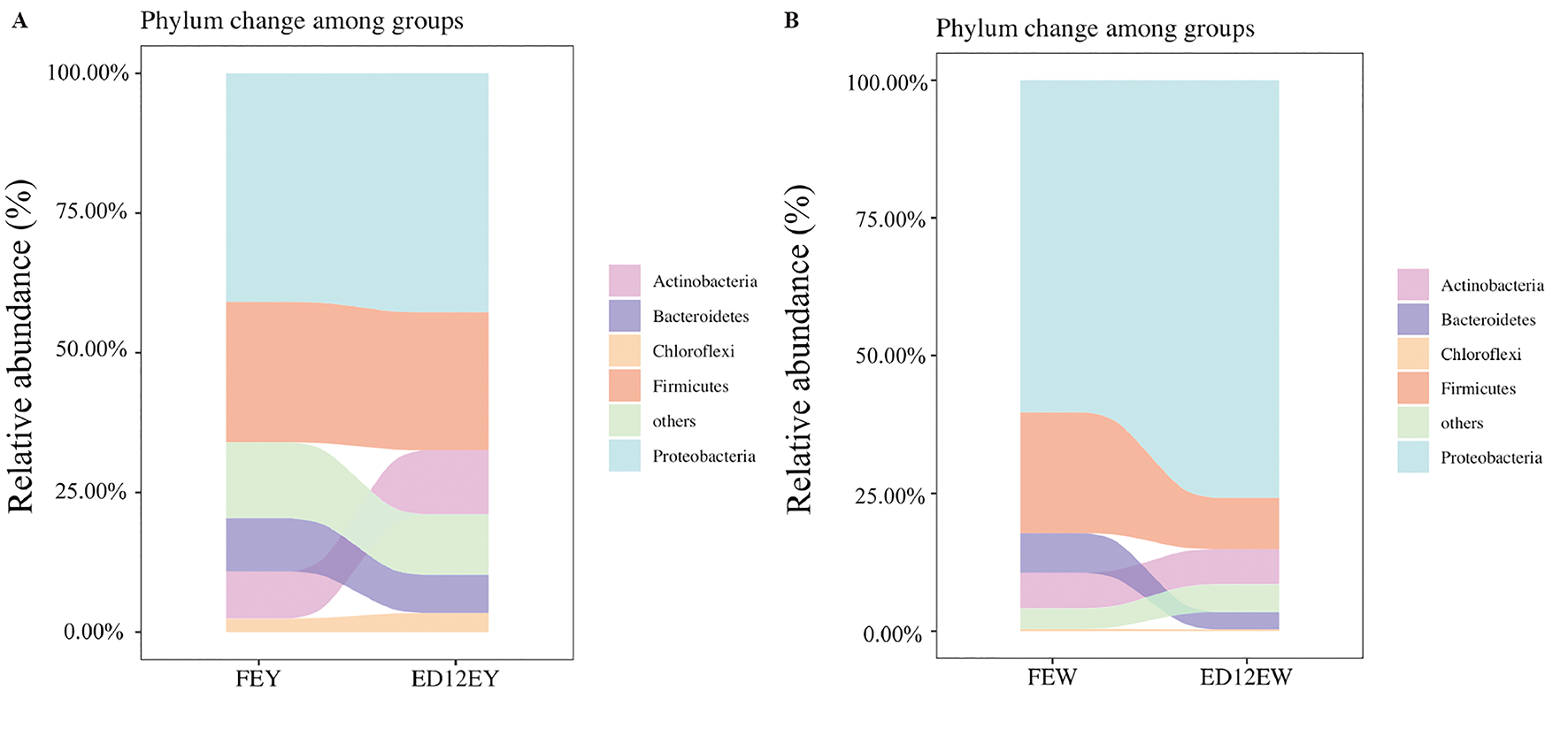

Supplement: Supplementary Figure 2 — (A) The alluvial diagram shows the results of the top 5 relative abundance of phyla in FEY and ED12EY. (B) The alluvial diagram shows the results of the top 5 relative abundance of phyla in FEW and ED12EW. [file Image_2.TIF]

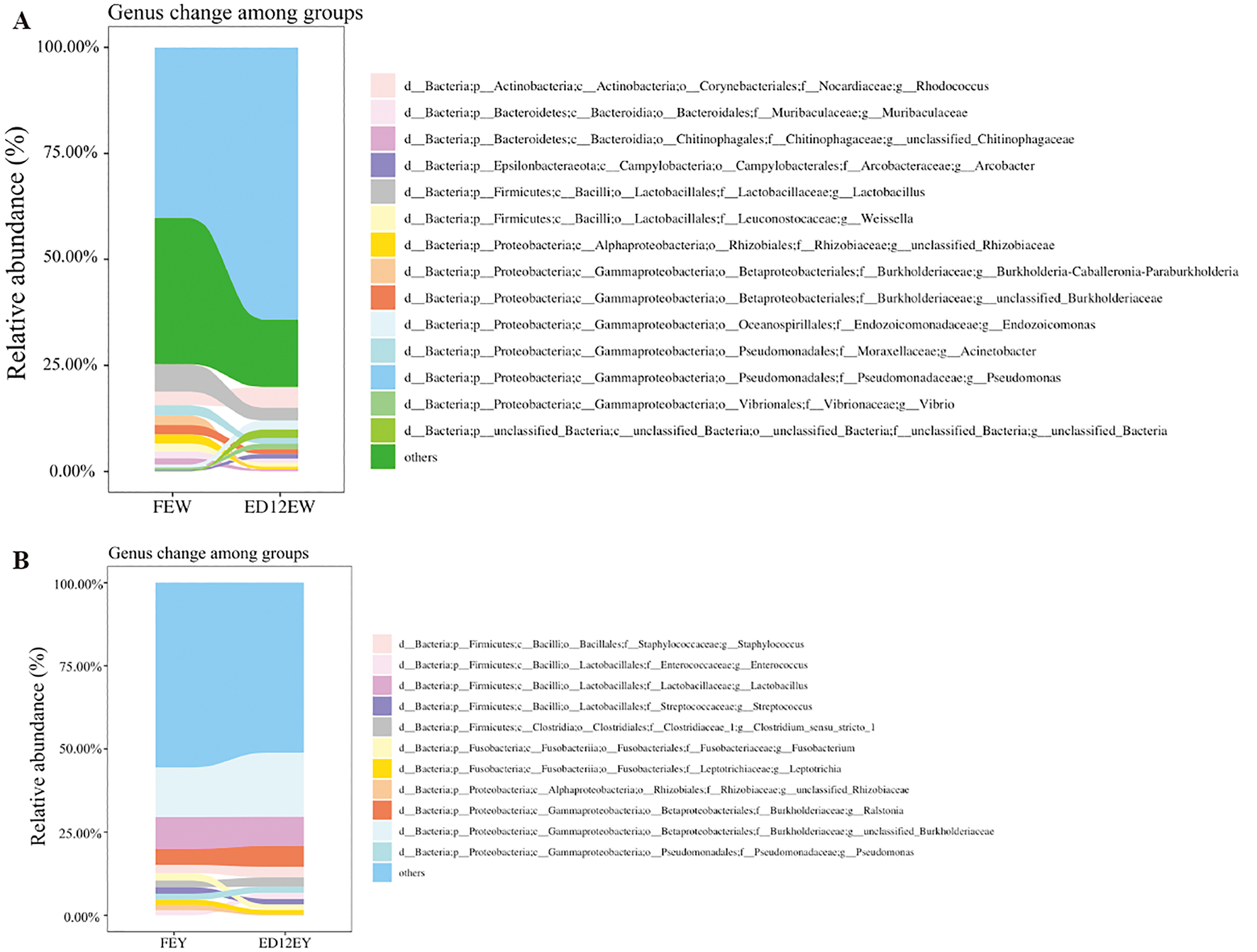

Supplement: Supplementary Figure 3 — (A) The alluvial diagram shows the results of the top 11 relative abundance of genera in four groups. The 11 genera were obtained by collecting the top 10 relatively abundance genera that did not overlap in FEY and ED12EY. (B) The alluvial diagram shows the results of the top 14 relative abundance of genera in four groups. The 14 genera were obtained by collecting the top 10 relatively abundance genera that did not overlap in FEW and ED12EW. [file Image_3.TIF]

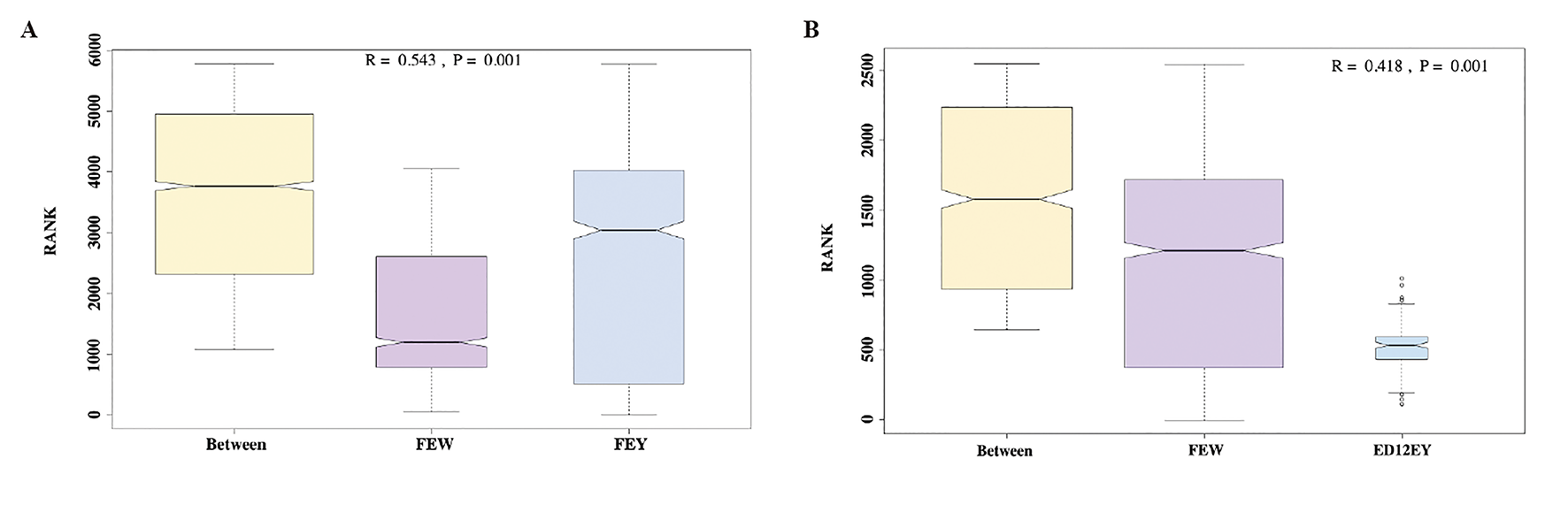

Supplement: Supplementary Figure 4 — (A) ANOSIM analysis between FEW and FEY. (B) ANOSIM analysis between FEW and ED12EY. [file Image_4.TIF]

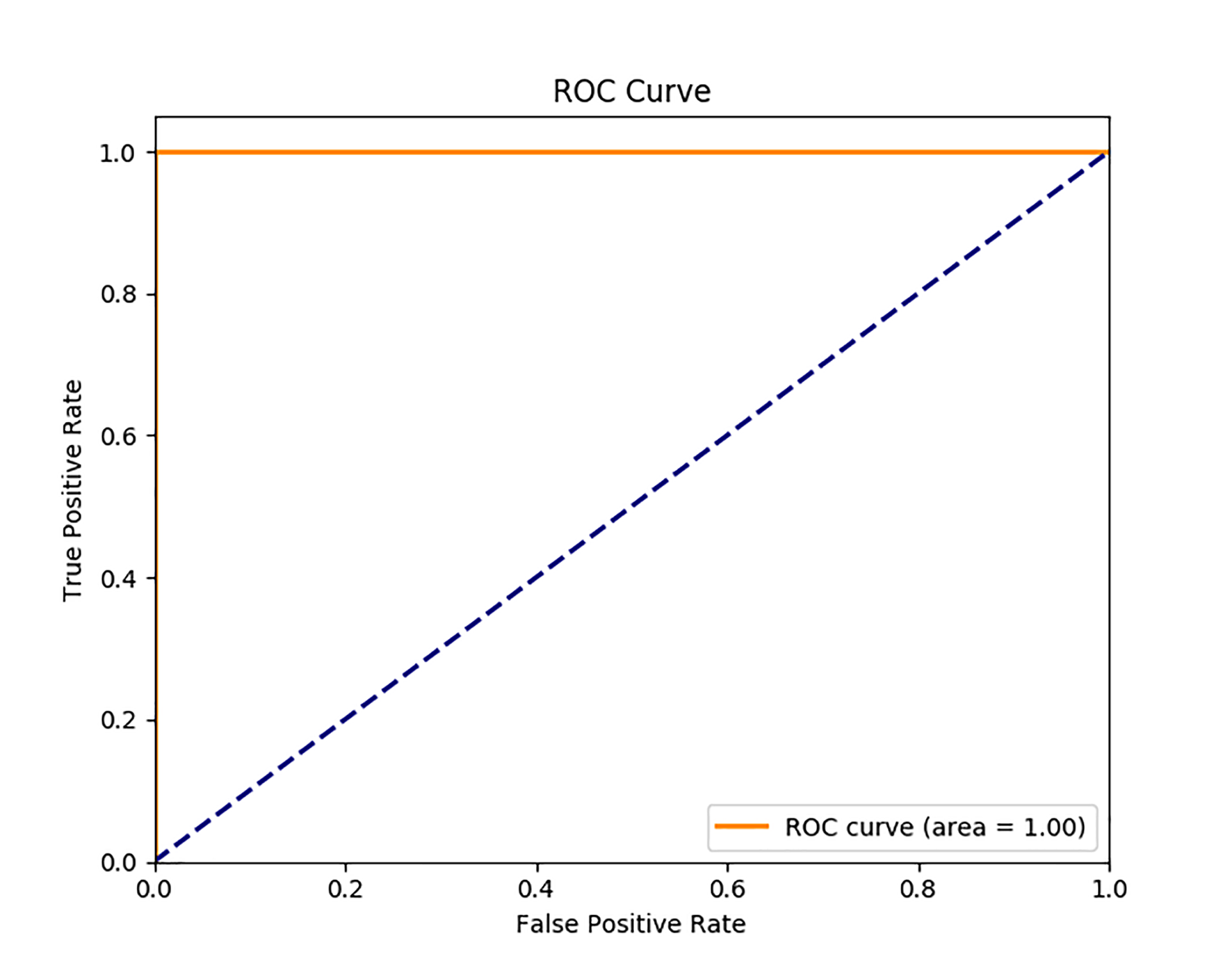

Supplement: Supplementary Figure 5 — Receiver operating characteristic (ROC) curve of Random Forest. [file Image_5.TIF]

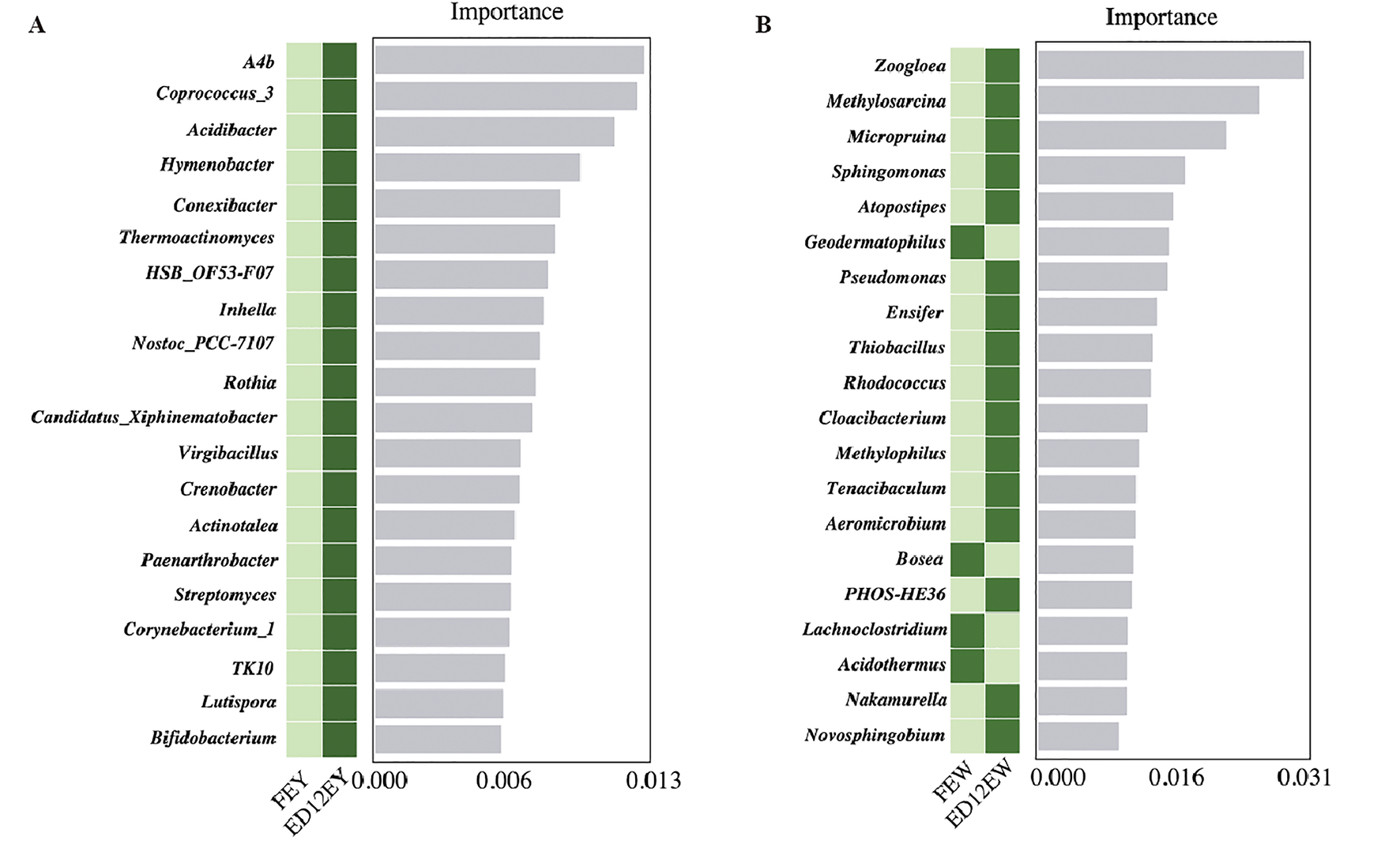

Supplement: Supplementary Figure 6 — (A) Random forest analysis identifies the key differential genera between FEY and ED12EY. (B) Random forest analysis identifies the key differential genera between FEW and ED12EW. [file Image_6.TIF]

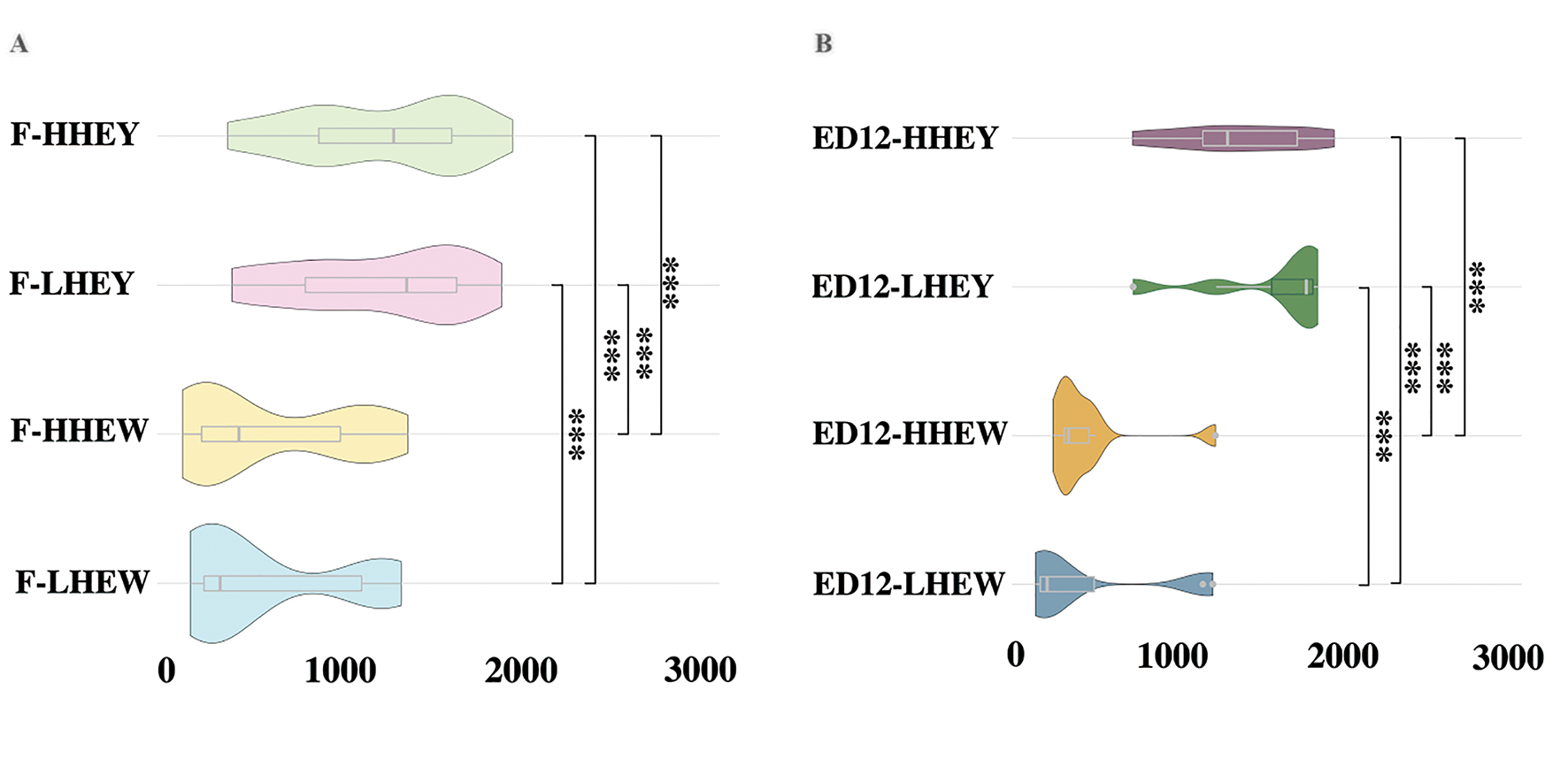

Supplement: Supplementary Figure 7 — Differences in the number of ASVs among egg yolks and eggs in high and low hatchability groups in fresh eggs and eggs for 12 days of incubation. [file Image_7.TIF]

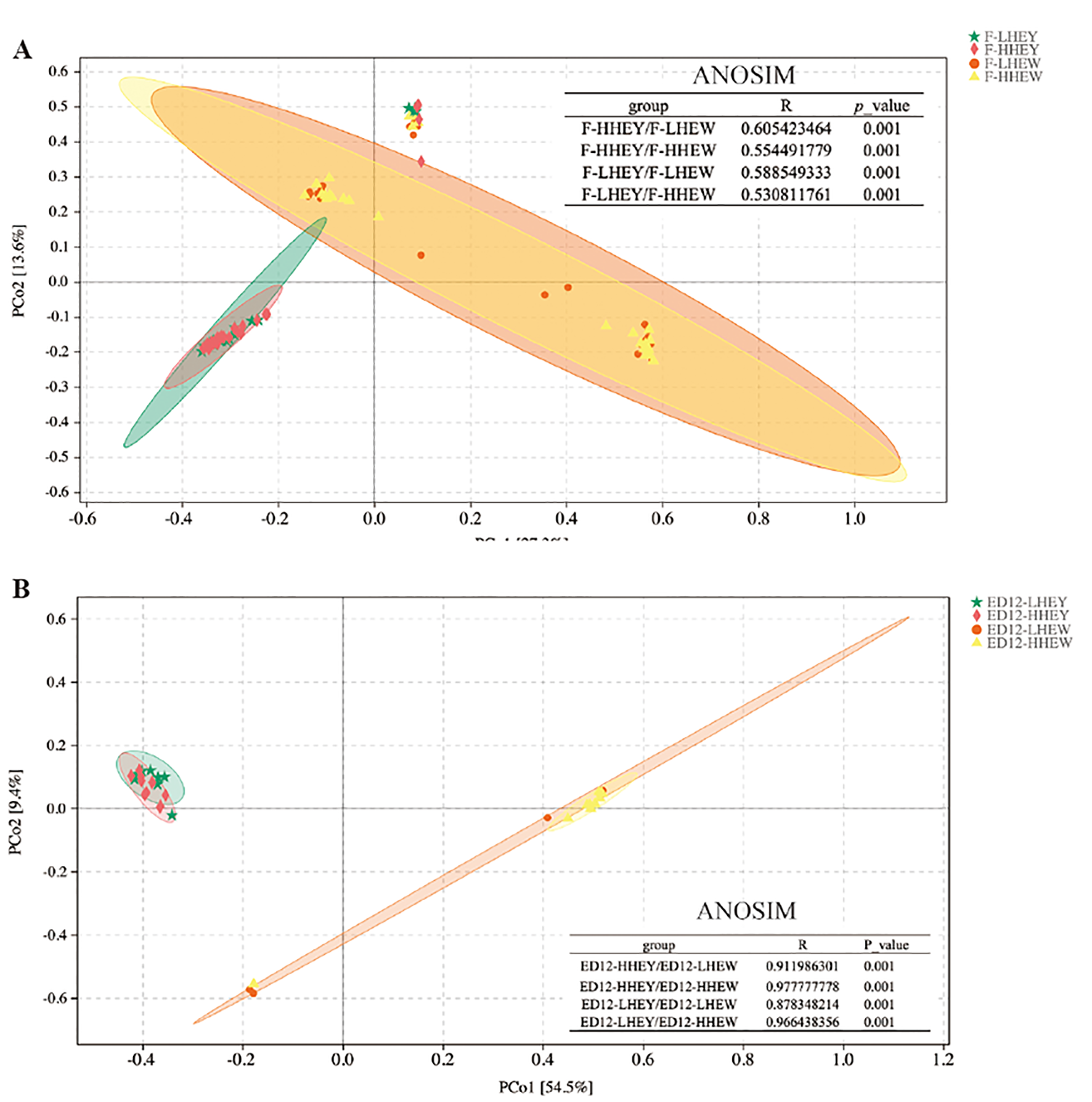

Supplement: Supplementary Figure 8 — (A) Principal coordinate analysis (PCoA) plot with ANOSIM table showed the similarity in the microbial composition of four groups of fresh eggs. (B) Principal coordinate analysis (PCoA) plot with ANOSIM table showed the similarity in the microbial composition of four groups of eggs after 12 days of incubation. [file Image_8.TIF]
